# Sucrose Consumption during Late Adolescence Impairs Adult Neurogenesis of the Ventral Dentate Gyrus without Inducing an Anxiety-like Behavior

**DOI:** 10.3390/ijms232214176

**Published:** 2022-11-16

**Authors:** Karla Sánchez-Huerta, Rosaura Debbie Saldaña-Salinas, Pablo Edson Bustamante-Nieves, Adriana Jiménez, Alejandro Corzo-Cruz, Marina Martínez-Vargas, Rosalinda Guevara-Guzmán, Iván Velasco, Enrique Estudillo

**Affiliations:** 1 Laboratorio de Neurociencias, Instituto Nacional de Pediatría, Insurgentes Sur 3700, Letra C, Coyoacán, Ciudad de México 04530, Mexico; 2 Laboratorio de Fisiología, Escuela Militar de Graduados de Sanidad, Secretaría de la Defensa Nacional, Batalla de Celaya 202, Lomas de Sotelo, Miguel Hidalgo, Ciudad de México 11200, Mexico; 3 Facultad de Ciencias, Universidad Nacional Autónoma de México, Ciudad de México 04510, Mexico; 4 Laboratorio de Reprogramación Celular, Instituto Nacional de Neurología y Neurocirugía Manuel Velasco Suárez, Insurgentes Sur 3877, La Fama, Tlalpan, Ciudad de México 14269, Mexico; 5 División de Investigación, Hospital Juárez de México, Mexico City 07760, Mexico; 6 Laboratorio Traslacional, Escuela Militar de Graduados de Sanidad, Secretaría de la Defensa Nacional, Batalla de Celaya 202, Lomas de Sotelo, Miguel Hidalgo, Ciudad de México 11200, Mexico; 7 Departamento de Fisiología, Facultad de Medicina, Universidad Nacional Autónoma de México, Circuito Exterior, Ciudad Universitaria, Ciudad de México 04510, Mexico; 8 Instituto de Fisiología Celular—Neurociencias, Universidad Nacional Autónoma de México, Circuito Exterior, Ciudad Universitaria, Ciudad de México 04510, Mexico

**Keywords:** ventral hippocampus, adult neurogenesis, ERK signaling, GABAergic system, immature granular neurons, KI67, Type 1 cells, Type 2b cells, Type 3 cells, neural stem cells

## Abstract

Sucrose consumption impairs behavioral and cognitive functions that correlate with decreased neurogenesis in animal models. When consumed during early adolescence, this disaccharide promotes anxious and depressive behaviors, along with a reduction in the generation of new neurons in the dentate gyrus of the hippocampus. Data concerning sucrose consumption during late adolescence are lacking, and the effect of sucrose intake on the ventral dentate gyrus of the hippocampus (which modulates anxiety and depression) remains elusive. Here, we tested whether sucrose intake during late adolescence causes anxiety or impaired neurogenesis in the ventral dentate gyrus. Rats did not display anxiety-like behaviors neither at the light–dark box test nor at the open field exploration. However, there was a significant increase in proliferative cells in the subgranular zone of the ventral dentate gyrus in rats exposed to sucrose (*p* < 0.05). This increased proliferation corresponded to neural stem cells (Radial Type 1 cells) in the group exposed to sucrose until adulthood but was not present in rats exposed to sucrose only during late adolescence. Remarkably, the phosphorylation of ERK1/2 kinases was increased in the hippocampi of rats exposed to sucrose only during late adolescence, suggesting that the increased proliferation in this group could be mediated by the MAPK pathway. On the other hand, although no differences were found in the number of immature granular neurons, we observed more immature granular neurons with impaired dendritic orientation in both groups exposed to sucrose. Finally, GAD65/67 and BCL2 levels did not change between groups, suggesting an unaltered hippocampal GABAergic system and similar apoptosis, respectively. This information provides the first piece of evidence of how sucrose intake, starting in late adolescence, impacts ventral dentate gyrus neurogenesis and contributes to a better understanding of the effects of this carbohydrate on the brain at postnatal stages.

## 1. Introduction

The hippocampus is a structure with prominent plasticity, where neurogenesis plays a role in this process [1]. Hippocampal neurogenesis is the mechanism through which new neurons are generated in the dentate gyrus (DG), and it is a sequential process where Type 1 cells (neural stem cells) divide and give rise to intermediate progenitors that differentiate into neuroblasts that express doublecortin (DCX), a marker also present in immature granular neurons (IGNs), which then progress to DCX-negative mature neurons [2]. The neurogenic process has been associated with cognitive and emotional processes, and its increase correlates with enhanced spatial memory as well as with anxiolytic and antidepressant effects [1,3,4,5,6,7]; several reports show that the generation of new neurons at the dorsal and ventral portions of the DG seems to be differentially affected by multiple factors, including stress, combination of inanimate and social stimulation (enriched environment), exercise, alcohol and drug consumption [6,7,8,9,10,11].

Diet also modulates hippocampal neurogenesis [12,13], but its differential effects at the neurogenic niches of the dorsal and ventral portions of the DG have remained understudied. In particular, the consumption of sucrose and fructose decreases neurogenesis, produces cognitive impairments and promotes an anxious and depressive-like phenotype when consumed during early adolescence in rats [14,15,16]. 

Adolescence is a period where the central nervous system undergoes deep changes in synaptic activity, dendritic pruning, growth of gray matter and myelinization in order to achieve complete brain maturation [17]. Notably, adolescence is a period where sucrose intake can produce long-term impairments in brain structures such as the hippocampus [15,16,18]. Although sucrose consumption during early adolescence promotes anxiety [16], it is unknown whether this impairment is also present when consumed during late adolescence, a period in which individuals are more susceptible to the consumption of several substances such as alcohol, drugs and added sugars [19,20,21]. Moreover, the impact of sucrose intake on the neurogenesis of the ventral DG has not been studied, even though this portion is associated with behavioral alterations including depression and anxiety [22,23]. Therefore, our main goal was to determine whether sucrose intake promotes anxiety or impairs neurogenesis in the ventral DG of rats after its consumption during late adolescence.

## 2. Results

### 2.1. Sucrose Treatments Do Not Modify Body Weight and Glycaemia

**Figure 1:**
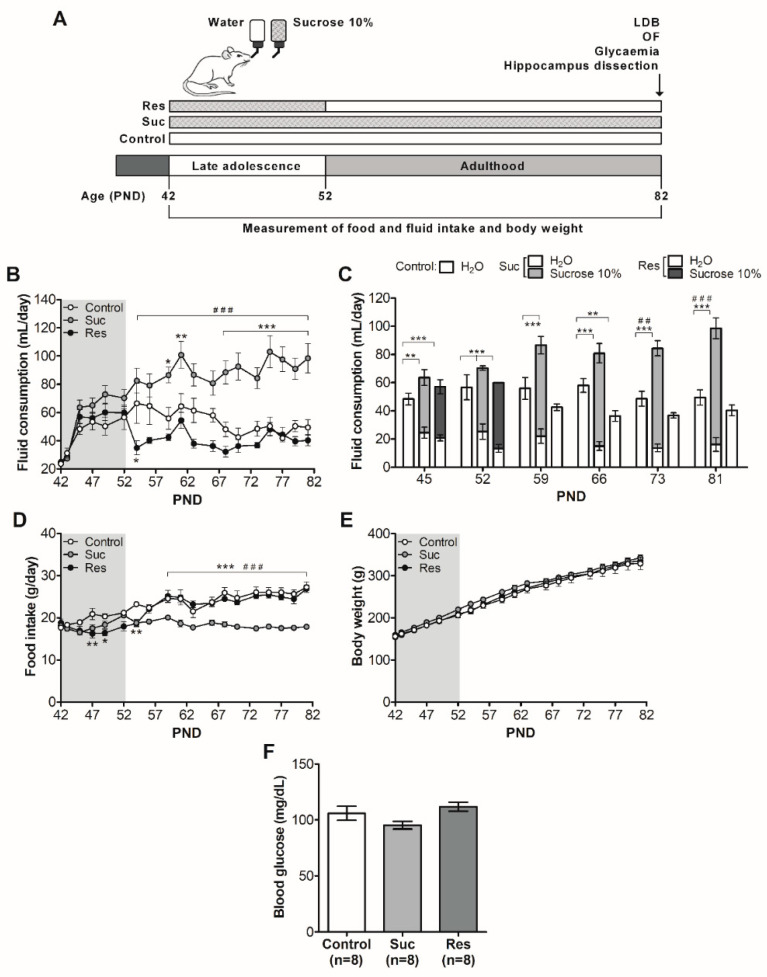
Effect of sucrose consumption on fluid and food intake, body weight and glycaemia. (**A**) Experimental design of the study. (**B**) Total fluid consumption in the experimental groups along the study. * *p* < 0.05, ** *p* < 0.01 and *** *p* < 0.001 Suc vs. control; ### *p* < 0.001 Suc vs. Res. (**C**) Water and sucrose consumption in the experimental groups. ** *p* < 0.01 and *** *p* < 0.001 water intake of the controls vs water intake of the sucrose exposed groups; ## *p* < 0.01 and ### *p* < 0.001 water intake of the controls vs sucrose intake of the Suc and Res groups. (**D**) Food intake along the experiment in all groups. * *p* < 0.05, ** *p* < 0.01 and *** *p* < 0.001 Suc vs. control; ### *p* < 0.001 Suc vs. Res. (**E**) The body weight of rats through the study. (**F**) The blood glucose of the experimental groups at PND 82. Repeated measures Two-Way ANOVA and Bonferroni tests were performed as statistical analysis for (**B**–**E**) (*n* = 8). One-Way ANOVA and Bonferroni tests were performed as statistical analysis for (**F**). The grey area in (**B**,**D**,**E**) represents the late adolescence period. LDB: light-dark box test; OF: open field test; PND: postnatal day.

To test whether sucrose consumption could induce either an anxious behavior or impaired neurogenesis when consumed during late adolescence, three different groups were designed: rats with access to only water (control), rats with access to sucrose from late adolescence to adulthood (Suc) and rats with access to sucrose only during late adolescence, followed by its restriction during adulthood (Res) (Figure 1A). We observed that the total amount of liquid intake (water and sucrose) increased in the Suc group when compared with that of the control group at PND 59, 61 and from PND 69 to PND 81, or that of the Res group from PND 54 to PND 81 (Interaction F_36,378_ = 5.876, *p* < 0.0001; Time F_18,378_ = 15.48, *p* < 0.0001; Treatment F_2,21_ = 20.73, *p* < 0.0001) (Figure 1B). Both groups exposed to sucrose preferred drinking sucrose over water, as previously reported [18]. The Suc group consumed less water than did the controls through the entire experiment; conversely, the volume of sucrose consumed in the Suc group was higher than the water volume consumed by the control group at PND 73 and 81. In the Res group, water intake was reduced during the sucrose exposure when compared with controls; however, after sucrose withdrawal, water consumption in the Res group was not different from the water consumption of the control group, with the exception of the PND 66 (Interaction F_20,175_ = 15.29, *p* < 0.0001; Fluid F_4,35_ = 49.06, *p* < 0.0001) (Figure 1C). Conversely, the Suc rats significantly reduced their food intake when compared to either the control or Res group from PND 59 to PND 81 (Interaction F_36,378_ = 8.527, *p* < 0.0001; Time F_18,378_ = 27.43, *p* < 0.0001; Treatment F_2,21_ = 21.96, *p* < 0.0001) (Figure 1D), which explains the absence of variations in the body weight between the three experimental groups throughout the entire study (Time F_18,378_ = 993.5, *p* < 0.0001) (Figure 1E). We also evaluated whether sucrose consumption could affect glycaemia; neither of the groups displayed abnormal levels when compared with the control rats (*p* = 0.0646) (Figure 1F).

### 2.2. Sucrose Consumption Starting at Late Adolescence Does Not Induce Anxiety

**Figure 2:**
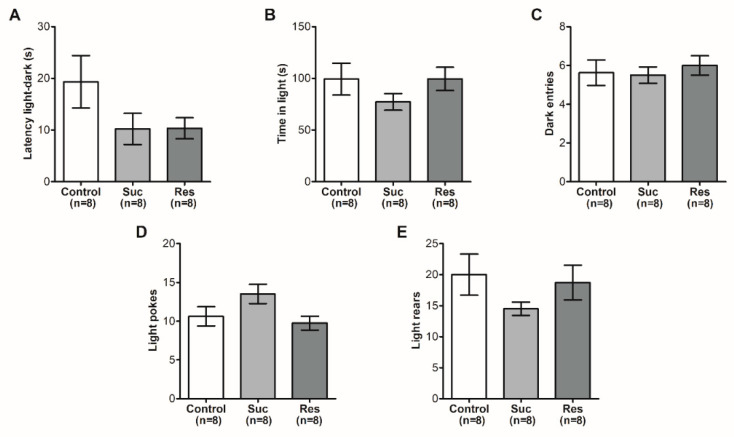
Sucrose exposure in late adolescence does not induce anxiety in the light–dark box test. (**A**) Latency to enter in the dark chamber. (**B**) Time spent in the light chamber. (**C**) Number of entries to the dark compartment. (**D**) Risk behavior measured by the number of pokes to light compartment. (**E**) Number of rears in the light chamber. These were analyzed via One-Way ANOVA followed by a Newman–Keuls test.

We evaluated anxiety behavior in the three groups at PND 82 using the light–dark box and open field tests. The Suc and Res groups did not display significant changes in the parameters analyzed after the light–dark box test (Latency *p* = 0.1481, time in light *p* = 0.3323, dark entries *p* = 0.7906, light pokes *p* = 0.0729 and rears *p* = 0.3077 (Figure 2)). Based on these results, it is unlikely that sucrose consumption during late adolescence could promote an anxious phenotype after reaching adult stages.

**Figure 3:**
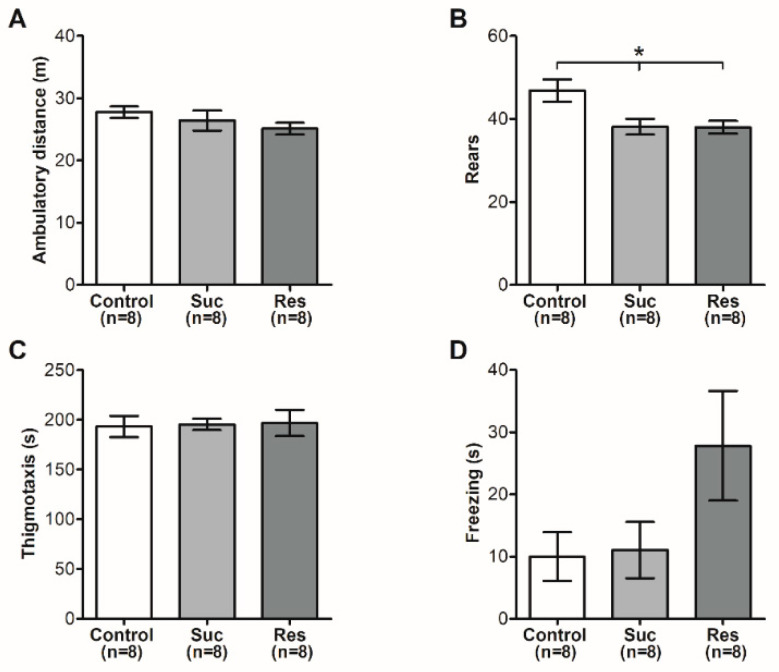
Sucrose consumption in late adolescence does not induce anxiety in the open field test. (**A**) Ambulatory distance traveled in the open field. (**B**) Number of rears in the arena. (**C**) Thigmotaxis or time traveled in the walls that enclose the area. (**D**) Time of immobility or freezing. These were analyzed via One-Way ANOVA followed by a Newman–Keuls test, * *p* < 0.05 vs. control.

Further data at the open field test supported the absence of an anxious phenotype, as no significant changes were observed in most of the conducts analyzed between the control group and those exposed to sucrose (Ambulatory distance *p* = 0.3227, thigmotaxis *p* = 0.9693 and freezing *p* = 0.0959) (Figure 3). Even though the Suc (38.13 ± 1.89) and Res (38.00 ± 1.52) groups showed a decreased rearing conduct when compared with the control group (46.88 ± 2.70, F_2,21_ = 5.880, *p* = 0.0094) (Figure 3B), this parameter is considered ambiguous when determining the presence of anxiety in the individuals analyzed [24]. Together, the results obtained in both tests suggest that rats in the late adolescent period are less susceptible to develop an anxious phenotype via sucrose consumption, in contrast to its intake during early adolescence [16,18].

### 2.3. Sucrose Intake during Late Adolescence Impaired Adult Neurogenesis in the Ventral DG

In order to know the effect of sucrose intake on adult neurogenesis in the ventral DG, we quantified several neurogenic populations cells, such as proliferative cells, proliferative radial and horizontal Type 1 cells, Type 2b/3 cells and neuroblasts (both of which were sorted into the same pool), IGNs and aberrant or ectopic IGNs. 

#### 2.3.1. Sucrose Intake during Late Adolescence Increases the Number of Proliferative Cells in the SGZ of the Ventral DG

**Figure 4:**
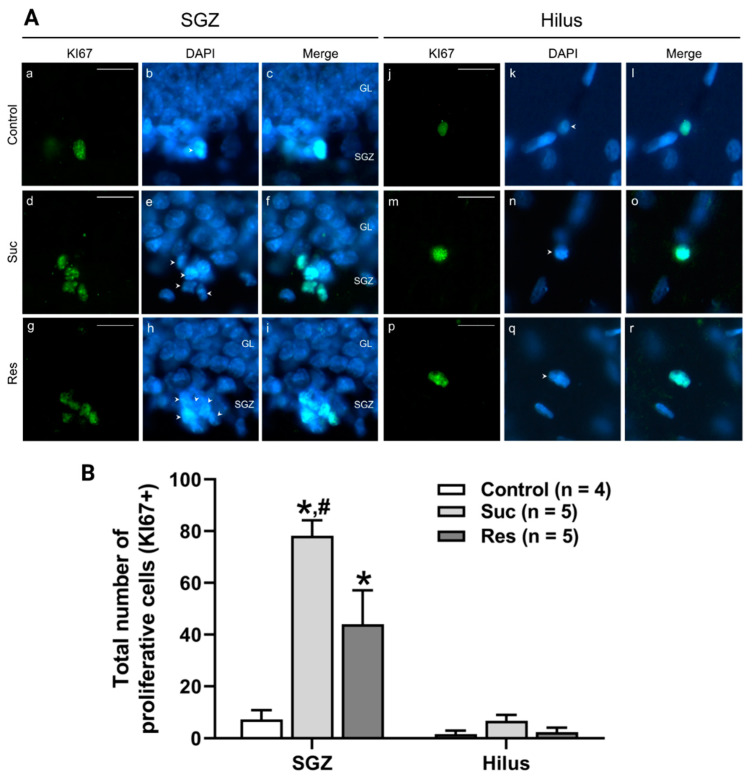
Sucrose consumption increases the number of proliferative cells (KI67+) in the subgranular zone of the ventral dentate gyrus. (**A**) Left: representative photomicrographs showing proliferative cells (KI67+, green), cell nuclei (DAPI, blue) and their merge in the subgranular zone (SGZ) of the ventral dentate gyrus in the control (a–c), Suc (d–f) and Res (g–i) groups. Right: representative photomicrographs of proliferative cells in the hilus of the ventral dentate gyrus in the control (j–l), Suc (m–o) and Res (p–r) groups. Arrowheads show the cell nuclei (DAPI+) that colocalized with KI67 marker. Note the abundance of proliferative cells in the SGZ of the ventral dentate gyrus in the Suc and Res groups. (**B**) Cell counting of proliferative cells located in the SGZ (left) or the hilus (right) of the ventral dentate gyrus. A higher number of proliferative cells were founded in the Suc and Res groups in comparison with the control group. Moreover, the number of proliferative cells was higher in the Suc group than in the Res group. No significant difference was found between groups when the hilus was analyzed. *: *p* < 0.05 vs control; #: *p* < 0.05 vs. Res. One-Way ANOVA followed by Tukey test. GL: granule cell layer. Scale bar: 20 µm.

To evaluate the effect of sucrose consumption on the mitotic phase of the adult neurogenesis of the ventral DG, we quantified the number of proliferative cells (KI67+) in the SGZ and the hilus. In the SGZ, the number of proliferative cells increased in the sucrose-treated groups with respect to the control group (Control: 7.3 ± 3.6, Suc: 78.2 ± 6.0; Res: 44 ± 13.1, One-Way ANOVA, Treatment: F_2,13_ = 14.266, *p* < 0.001. Tukey test: *p* < 0.001 Suc vs. control and *p* = 0.045 Res vs. control). Moreover, there was a higher number of proliferative cells in the Suc group versus the Res group (*p* = 0.048). In the hilus, there were no significant differences between the analyzed groups (Control: 1.6 ± 1.3, Suc: 6.8 ± 2.3; Res: 2.4 ± 1.7, One-Way ANOVA, Treatment: F_2,14_ = 2.385, *p* = 0.134) (Figure 4).

#### 2.3.2. Sucrose Consumption during Late Adolescence Increases the Number of Proliferative Type 1 Cells in the SGZ of the Ventral DG

**Figure 5:**
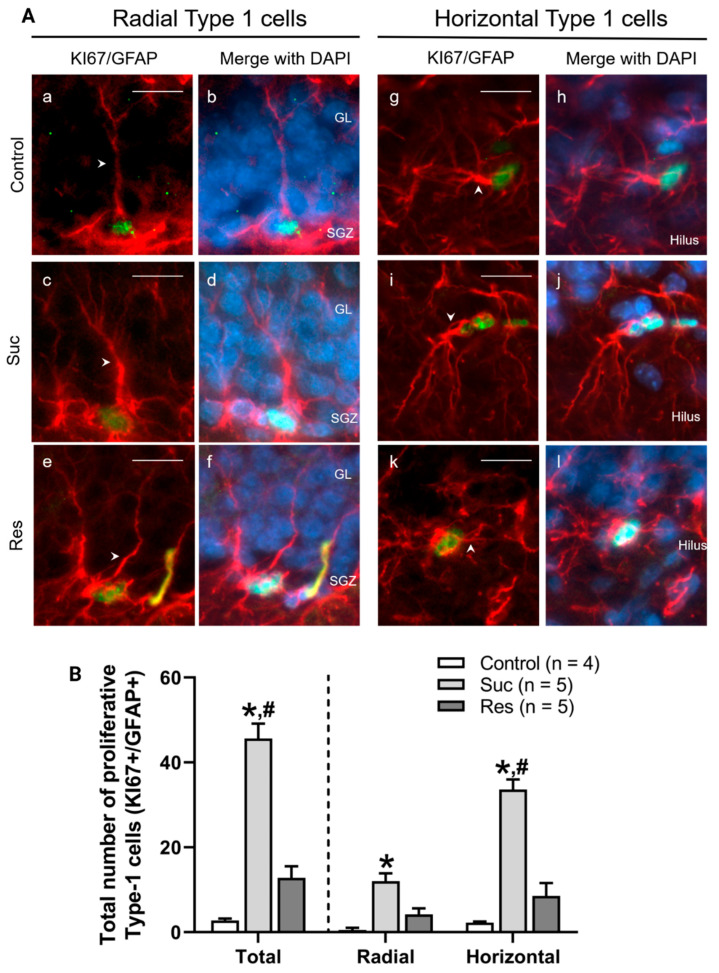
Sucrose consumption during late adolescence increases the number of Radial and Horizontal Type 1 cells in the subgranular zone of ventral dentate gyrus. (**A**) Representative photomicrographs of proliferative Type 1 cells (KI67+/GFAP+) located in the subgranular zone (SGZ) of the ventral dentate gyrus (DG). Left: proliferative Radial Type 1 cells (KI67: green, GFAP: red) and their merge with DAPI (blue) in the SGZ in the control (a,b), Suc (c,d) and Res (e,f) groups. The arrowhead indicates the dendritic arborization showing their radial morphology. Right: proliferative horizontal Type 1 cells and their merge with DAPI in the SGZ in the control (g,h), Suc (i,j) and Res (k,l) groups. The arrowhead indicates the dendritic projections showing their horizontal morphology. (**B**) A cell counting of proliferative Type 1 cells located in the SGZ of the ventral DG. Left: a cell counting of the total number of proliferative Type 1 cells in the SGZ of the control, Suc and Res groups. The number of these cells in the Suc group was higher than those in the control and Res groups. Right: a cell counting of the subpopulations of Type 1 cells (radial and horizontal) in the SGZ of the control, Suc and Res groups. The Suc group showed an increased number of both Radial and Horizontal Type 1 cells in comparison with the control group. Moreover, this group exhibited a higher number of horizontal cells versus the Res group. *: *p* < 0.05 vs control, #: *p* < 0.05 vs Res. Total and Horizontal Type-1 cells were analyzed via One-Way ANOVA followed by a Tukey test. Radial Type 1 cells were analyzed by a Kruskall Wallis Test followed by a Dunn’s test. GL: granule cell layer. Scale bar: 20 µm.

To assess the effect of sucrose consumption on the neural stem cells of the ventral DG, we quantified the number of proliferative Type 1 cells (GFAP+/KI67+) in the SGZ of the ventral DG. The total number of proliferative Type 1 cells augmented in the Suc group was compared with those of the control group (control: 2.75 ± 0.5, Suc: 45.6 ± 3.5; Res: 12.8 ± 2.7, One-Way ANOVA, Treatment F_2,11_ = 64.67, *p* < 0.001. Tukey test: *p* < 0.001 Suc vs. control). Moreover, a higher number of proliferative Type 1 cells was found in the Suc group versus the Res group (*p* < 0.001) (Figure 5B-left). 

In agreement with the morphological criteria, we identified two subpopulations of Type 1 cells: radial and horizontals. The Radial Type 1 cells subpopulation was augmented in the Suc group when compared with that in the control group (Control: 0.5 ± 0.5, Suc: 12 ± 1.9, Res: 4.2 ± 1.4, Kruskal–Wallis Test, Treatment H(2) = 10.365, *p* < 0.01. Dunn’s test: *p* < 0.05 Suc vs. control). The number of horizontal Type 1 cells also increased when compared with the control group (Control: 2.2 ± 0.2, Suc: 33.6 ± 2.4 8; Res: 8.6 ± 3.0, One-Way ANOVA, Treatment F_2,11_ = 48.362, *p* < 0.001. Tukey test: *p* < 0.001 Suc vs control). Moreover, the number of horizontal Type 1 cells increased in the Suc group versus the Res group (*p* < 0.001) (Figure 5B-right). 

#### 2.3.3. Sucrose Consumption Leads to Alterations in the Population of DCX-Immunoreactive Cells

**Figure 6:**
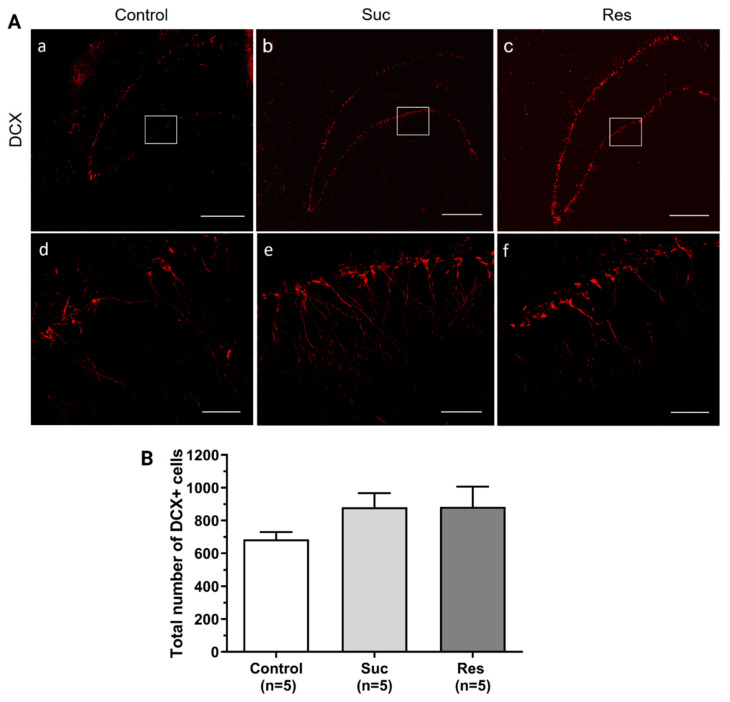
Sucrose consumption does not modify the total number of doublecortin (DCX)-immunoreactive cells in the ventral dentate gyrus. (**A**) Representative confocal photomicrographs showing the presence of DCX+ cells (red) in the ventral dentate gyrus in the control (a), Suc (b) and Res (c) groups. A higher magnification of the zones enclosed within white boxes is shown for the control (d), Suc (e) and Res (f) groups. (**B**) Cell counting of the total DCX+ cells located in the dentate gyrus of the ventral hippocampus. Although the Suc and Res groups showed a tendency to increase the total number of DCX+ cells, no significant differences were found. One Way ANOVA. Scale bar (a–c): 500 µm, scale bar (d–f): 50 µm.

We found that the total number of DCX+ cells was not significantly different among the groups (Control: 685.8 ± 43.93, Suc: 881.4 ± 85.89, Res: 883.4 ± 123.04, One-Way ANOVA, Treatment: F_2,12_:1.581, *p* = 0.246) (Figure 6). Despite the lack of effect of sucrose consumption on the overall population of DCX+ cells, we wanted to investigate whether subpopulations of DCX+ cells were selectively affected by this disaccharide. For this, we identified various cell types, including a pool of Type 2b/3 cells and neuroblasts, IGNs and aberrant or ectopic IGNs. 

**Figure 7:**
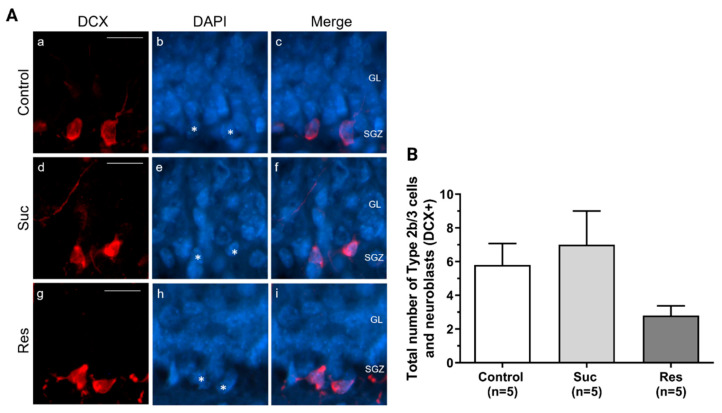
Sucrose consumption does not modify the pool of Type 2b/3 cells and neuroblasts in the subgranular zone of the ventral dentate gyrus. (**A**) Representative photomicrographs showing Type 2b/3 cells and neuroblasts (DCX+, red), and their merge with DAPI (blue) in the subgranular zone (SGZ) of the ventral dentate gyrus (DG) in the control (a–c), Suc (d–f) and Res (g–i) groups. Arrowheads show the cell nuclei (DAPI+) that colocalized with DCX marker. (**B**) A cell counting of Type 2b/3 cells and neuroblasts located in the ventral DG. No significant difference was found. Kruskall Wallis test. GL: granule cell layer. Scale bar: 20 µm.

**Figure 8:**
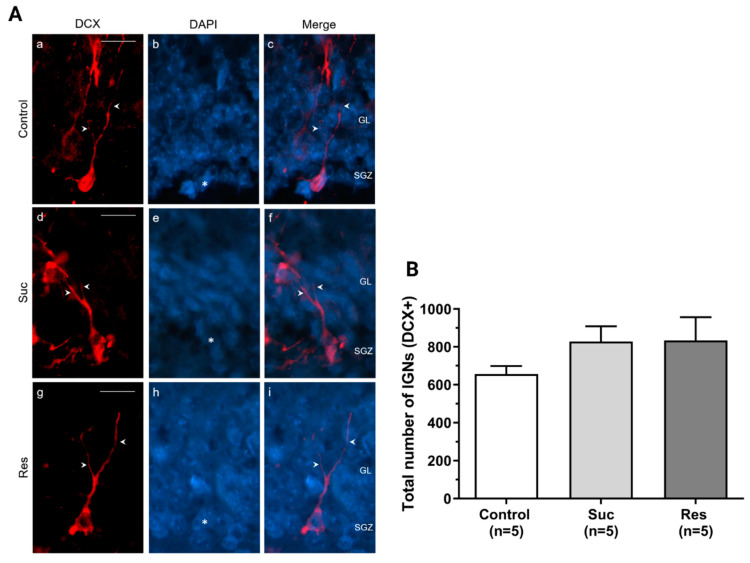
Sucrose consumption does not modify the number of immature granular neurons (IGNs) in the subgranular zone of the ventral dentate gyrus. (**A**) Representative photomicrographs showing the presence of IGNs (DCX+, red) and their merge with DAPI (blue) in the subgranular zone (SGZ) of ventral dentate gyrus (DG) in the control (a–c), Suc (d–f) and Res (g–i) groups. The arrowheads indicate the dendritic arborization of the IGNs typically oriented toward the granule cell layer (GL) of the DG. Arrowheads show the cell nuclei (DAPI+) that colocalized with KI67 marker. (**B**) A cell counting of IGNs located in the SGZ of the ventral DG. No significant difference was found. One-Way ANOVA. Scale bar: 20 µm.

We did not observe significant differences either in the total number of Type 2b/3 cells and neuroblasts (Control: 5.80 ± 1.28, Suc: 7.0 ± 2.0, Res: 2.8 ± 0.58, Kruskal Wallis test, Treatment H(2) = 4.251, *p* = 0.119) or in the amount of IGNs (Control: 656.2 ± 42.8, Suc: 828.2 ± 80.16, Res: 833.6 ± 123.82, One-Way ANOVA, Treatment: F_2,12_:1.295, *p* = 0.310) (Figure 7 and Figure 8, respectively).

**Figure 9:**
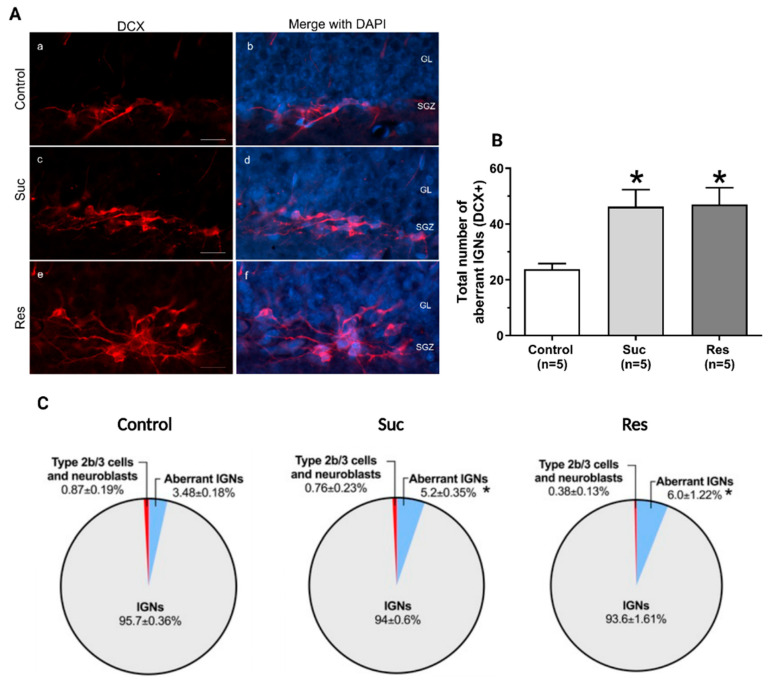
Sucrose consumption during late adolescence increases the number and proportion of aberrant immature granular neurons (IGNs) in the subgranular zone of the ventral dentate gyrus. (**A**) Representative photomicrographs of aberrant IGNs (DCX+, red) and their merge with DAPI (blue) in the subgranular zone (SGZ) of the ventral dentate gyrus (DG) in the control (a,b), Suc (c,d) and Res (e,f) groups. Note the abundance of aberrant IGNs in the Suc and Res groups. GL: granule cell layer. Scale bar: 20 µm. (**B**) Cell counting of aberrant IGNs in the SGZ of ventral DG of the control, Suc and Res groups. A significant increase in aberrant IGNs was found in the Suc and Res groups when compared with the control group. *: *p* < 0.05 vs. control. One-Way ANOVA followed by a Tukey test. (**C**) Pie charts showing the composition of the pool of doublecortin-immunoreactive cells in the SGZ of the ventral DG in the control, Suc and Res groups. In the control group, the pool of DCX+ cells was constituted mainly by IGNs, followed by Type 2b/3 cells and neuroblasts. In this group, the percentage of aberrant IGNs was about 3.5%. Interestingly, the sucrose-treated groups showed a significant increment in the percentage of aberrant IGNs (about 5–6%). *: *p* < 0.05 vs percentage of aberrant IGNs in control group. We used a Kruskal Wallis test followed by a Tukey test.

**Figure 10:**
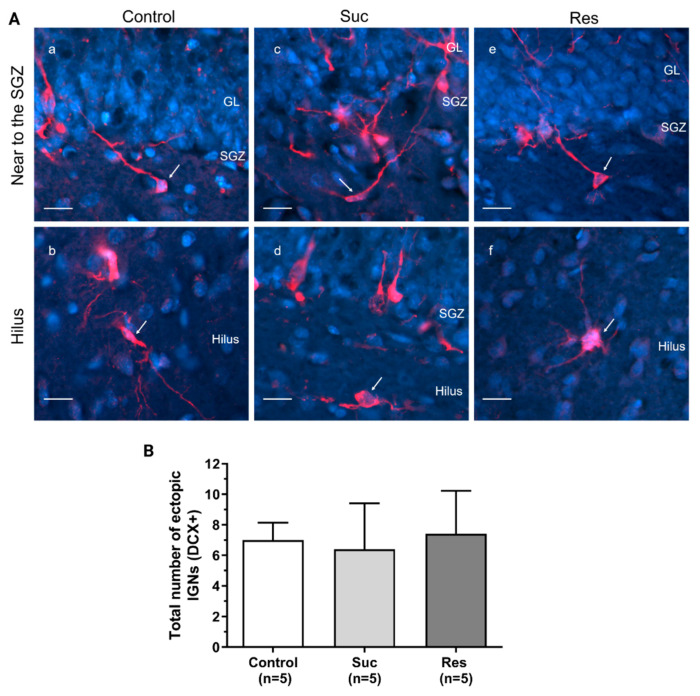
Sucrose consumption during late adolescence does not affect the number of ectopic immature granular neurons (IGNs) in the ventral dentate gyrus. (**A**) Representative photomicrographs of ectopic IGNs (DCX+, red) merged with DAPI (blue) and located near to the subgranular zone (SGZ) or in the hilus in the control (a,b), Suc (c,d) and Res (e,f) groups. The arrows indicate ectopic IGNs. Note that IGNs with ectopic distribution are present not only in sucrose-treated groups but also in the control group. GL: granule cell layer. Scale bar: 20 µm. (**B**) A cell counting of ectopic IGNs in the ventral dentate gyrus of the control, Suc and Res groups. No significant difference was found among the experimental groups. One-Way ANOVA.

Interestingly, we found that the sucrose-treated groups showed a significant increase in the total number of aberrant IGNs with respect to the control group (Control: 23.8 ± 2.08, Suc: 46.2 ± 6.11, Res: 47 ± 6.06, One-Way ANOVA, Treatment: F_2,12_:6.638, *p* = 0.011. Tukey test: *p* = 0.019 Suc vs control and *p* = 0.023 Res vs control) (Figure 9A-B). Moreover, it was found that the sucrose-treated groups exhibited a pool of DCX+ cells with a higher proportion of aberrant IGNs versus the control group (Control:3.48 ± 0.18%, Suc: 5.2± 0.35%, Res: 6.0 ± 1.22%, Kruskal Wallis Test, H(2) = 8.503, *p* = 0.04. Tukey test: *p* < 0.05 Suc vs control and *p* < 0.05 Res vs control) (Figure 9C). Finally, we did not find significant differences in the number of ectopic IGNs among the Suc and Res groups (One Way ANOVA, F_2,12_= 0.0413, *p* = 0.960) (Figure 10). 

### 2.4. Sucrose Consumption Increases Phospho-Erk1/2 Levels in the Hippocampus of Rats Exposed to Sucrose Only during Late Adolescence

**Figure 11:**
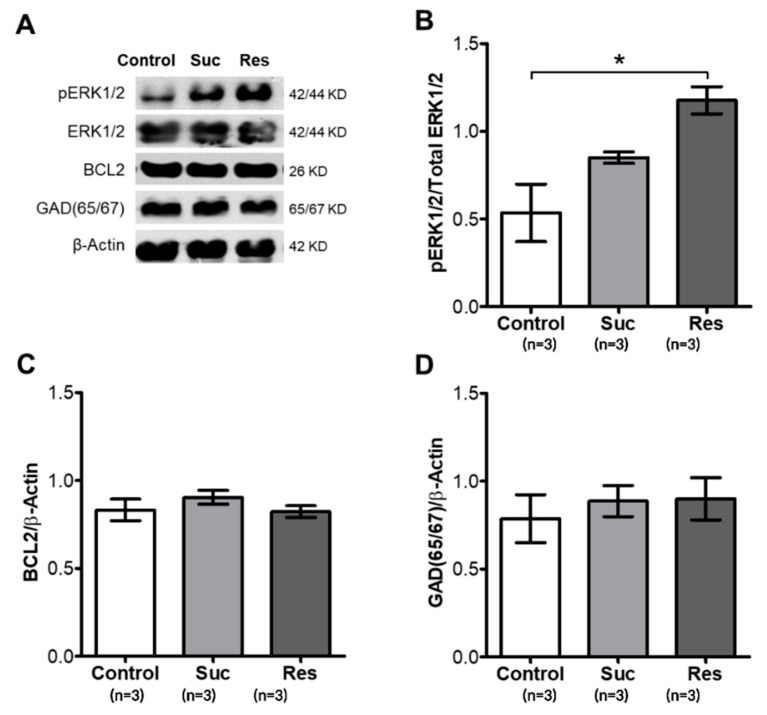
Sucrose exposure restricted to the late adolescence increases ERK1/2 phosphorylation in the hippocampus. (**A**) Representative images of Western blots for protein analyzed. (**B**) The densitometry of phosphorylated levels of ERK1/2, normalized by total ERK1/2. (**C**) BCL2. (**D**) Glutamate Decarboxylase (GAD65/67) in all the experimental groups. One-Way ANOVA and Tukey test, * *p* < 0.05.

To investigate the mechanisms that could be mediating progenitor proliferation, we tested whether pathways related with proliferation were modified in the Suc and Res groups. Western blot assays from the whole hippocampal formation were performed to examine the phosphorylation levels of the downstream effector of the MAPK pathway ERK1/2 as well as the antiapoptotic protein BCL2. We found a significant increase in phospho-ERK1/2 in the Res group (1.177 ± 0.077) and a tendency for it to increase in the Suc group (0.849 ± 0.0325) when compared with the control group (0.534 ± 0.164, F_2,6_ = 9.088, *p* = 0.0153) (Figure 11A,B), suggesting that progenitor proliferation could be mediated by the MAPK pathway. On the other hand, the antiapoptotic protein BCL2 did not show different expression levels between the three groups analyzed (*p* = 0.4449) (Figure 11A,C), suggesting that antiapoptotic processes are not related to the increase in progenitor proliferation after sucrose intake. Finally, since previous studies demonstrated that the GABAergic system is impaired in the cortex but not the hippocampus after sucrose intake during early adolescence [18], we decided to analyze the Glutamate Decarboxylase (GAD65/67) expression, which is a broad marker for inhibitory interneurons. Interestingly, the three groups displayed similar levels of GAD 65/67, with no differences between them (*p* = 0.7642) (Figure 11A,D).

## 3. Discussion

Sucrose consumption impairs cognitive functions and adult neurogenesis and induces anxious and depressive-like phenotypes [15,16,18,25]. Our study describes the effect of short- and long-term access to sucrose when its intake starts at late adolescence through the Res and Suc groups, respectively. Herein, we demonstrated that sucrose intake does not produce anxiety in rats when its consumption is either restricted to late adolescence or allowed from late adolescence to adulthood. These results contrast from those reported before, where sucrose intake spanned only the early adolescent stage and produced anxiety [16]. Other studies have shown, however, that sucrose intake performed during adulthood did not produce anxious behavior [16,26]. Therefore, along with previously reported data, our results suggest that it is likely that late adolescent rats display a phenotype more related to adult individuals than to early adolescent subjects and, as they reach adulthood, the anxiogenic effects of sucrose are lost. Interestingly, depression has also been associated with individuals that were deprived of sucrose [16,25]. Determining the impact of sucrose withdrawal on depression would provide valuable data, since this behavioral state was not measured in this study and further research must be performed to characterize the complete effect of sucrose consumption on late adolescent stages. 

Even though we did not find an anxious behavior in the Suc and Res groups, there was an increase in the total number of proliferative cells at the ventral DG. The physiological relevance of the augmented proliferation on the DG after sucrose consumption remains to be unveiled; however, our results highlighted that the proliferative pattern is distinct between the Suc and Res groups. The increase of either radial or horizontal Type 1 cells in the Suc group only highlights the effect of sucrose intake during long periods (more than 30 days) on the neural stem cells and suggests that either glial or neural progenitor pools could increase in this condition. Whether these changes also depend on the period (adolescence or adulthood) at which sucrose consumption began remains to be unveiled. On the other hand, the total number of proliferative cells was increased in the Res group; however, the proliferation of horizontal and radial Type 1 cells did not change. Therefore, the increase in proliferation in this experimental group could involve other cell types in the neurogenic process (such as Type 2a cells or astrocytes). 

The fact that sucrose consumption induced an increase in cell proliferation at the DG suggests the activation of signaling pathways related with this process. In line with this notion, there was a significant increase in phospho-ERK1/2 in the Res group when compared with controls, which suggests that MAPK pathways could be promoting cell proliferation in this group. Conversely, the unchanged levels of phospho-ERK1/2 of the Suc group suggest that the ERK1/2 pathway is not relevant for the proliferation of Type 1 cells in a long-term exposure to sucrose and other kinases related to survival and proliferation pathways should be explored, such as AKT [27]. Moreover, it must be considered that phospho-ERK1/2 could be increased as a result of the sucrose withdrawal in the Res group, since the phosphorylation of ERK1/2 increases after the withdrawal of a stimulus such as exercise [28]. Alternatively, phospho-ERK1/2 could be involved in other functions not related with cell survival and proliferation, since the ERK1/2 pathway also modulates neuronal plasticity in the hippocampus; specifically, it is required for long term potentiation (LTP) [29]. Therefore, the ERK1/2 pathway could be modulating plastic processes.

The molecular cues that promote the increase of proliferation and ERK1/2 phosphorylation remain unknown; it is likely that some neurotrophins such as BDNF or NGF could be promoting cell proliferation and shall be explored in a near future [8,30,31]. Although it has been demonstrated that BCL2 promotes neuronal survival [32], sucrose intake did not increase BCL2 levels, which suggests that the increased number of proliferative cells might not be dependent on BCL2. Other antiapoptotic proteins shall be investigated to determine their contribution to cell proliferation after sucrose intake.

Although sucrose-treated rats did not exhibit an increased population of IGNs when compared with controls, the presence of increased aberrant IGNs suggest cellular impairments that could compromise cognitive functions. Interestingly, the number of IGNs with altered orientation was not different between the Suc and Res groups, suggesting that sucrose intake during late adolescence induces permanent changes in dendritic arborization that remain at adult stages. Even though increased neurogenesis has been related to beneficial factors such as exercise, enriched environment or antidepressant treatments, it has also been linked to other unfavorable conditions [1,6]. Previous reports showed an increase in IGNs in rats that were exposed to social stress during early adolescence [33]. Further evidence demonstrated that rodents with seizures also demonstrated an increased number of newborn neurons during adulthood; however, these young cells displayed an aberrant morphology and distribution [34,35]. Therefore, it is important to highlight the increased number of IGNs with altered orientation of their dendritic processes after sucrose consumption and a deeper analysis of the morphology, connectivity and behavior should be performed in order to determine whether augmented neurogenesis due to sucrose consumption during late adolescence results in beneficial or detrimental effects to the nervous system.

The unaffected hippocampal levels of GAD65/67 in the Suc and Res groups suggest an unchanged hippocampal GABAergic system after sucrose intake, which supports the absence of anxiety. However, it remains to be determined whether there is a change in parvalbumin (PV) interneurons after sucrose intake [15]. These results support the absence of an anxious phenotype in sucrose-treated rats, since this could suggest an unchanged population of PV GABAergic interneurons of the DG, which negatively modulate anxiety [36]; however, PV interneurons should be analyzed in further studies. 

Until now, studies focused on the impact of sucrose consumption on neurogenesis were limited to the dorsal DG or the entire structure without differentiating between its dorsal and ventral portion [14,16]. Therefore, no information concerning to the ventral DG had been documented. We provide the first evidence of the way sucrose intake modulates neurogenesis at the ventral DG during late adolescence.

Although the aim of the present report was the study of the neurogenesis at the ventral DG, it is also important to address, in the same conditions, the impact of sucrose consumption during late adolescence on the neurogenesis at the dorsal dentate gyrus. Moreover, it could be relevant to determine whether there are tasks modulated by this hippocampal portion that could also be affected by sucrose consumption after reaching adult stages such as spatial memory [22]. Finally, it must be considered that other behavioral tests such as the elevated plus maze or novelty suppressed feeding tests were not performed in this study and could further support the lack of an anxious phenotype [16,37]. Additionally, the present study did not evaluate newborn mature neurons identified via colabeling NeuN/BrdU positive neurons [16,38]. Therefore, more studies should be performed to determine the complete contribution of new neurons generated during the period of sucrose intake.

## 4. Methods and Materials

### 4.1. Animals and Treatments

Animal handling and procedures were approved by the Instituto de Fisiología Celular-UNAM Animal Care and Use Committee (Protocol IVV168-20) and by the Instituto Nacional de Pediatría Animal Care and Use Committee (Protocol 2021/C037) in agreement with local (NOM-062-ZOO-1999) and international guidelines (Guide for the Care and Use of Laboratory Animals of the National Institutes of Health). Twenty-four male Wistar rats were kept in a 12/12 h light/dark cycle with free access to standard food pellets and water before and during the entire experiment. On postnatal day 42 (PND 42) (which is considered late adolescence [39], rats were randomly assigned to one of three different groups: (i) the control group with free access to two bottles with water; (ii) the sucrose group (Suc) with free access to a bottle with water and a bottle with a 10% sucrose solution from late adolescence (PND 42) to adulthood (PND 82); and (iii) the restricted group (Res) a group with free access to water and 10% sucrose solution but only during the late adolescence period (PND 42-PND 52)—after this period, the sucrose was withdrawn and replaced with water until adulthood stages (PND 82). Fluid and food consumption, as well as body weight, were measured through the entire experiment (PND 42-82). Fluid and food consumption were measured by registering the volume of liquid and the weight of food before and after its consumption every three days. Every three days, the differences between the values obtained for liquid (volume) and food (weight) before and after their consumption were divided in two and graphed. Blood glucose levels were determined at PND 82 from a blood drop obtained from the tail vein of nonfasted rats using an Abbott glucometer. 

### 4.2. Behavioral Testing

After each treatment, at PND 82, rats were submitted to the light/dark box followed by the open field tests to determine the presence of an anxiety-like behavior. Both tests were performed during the light cycle.

#### 4.2.1. Light/Dark Box Test

Preferences for dark or light compartments in a light–dark box were assessed in a rectangular acrylic box consisting of a 30 × 30 × 30 cm^3^ (long × width × high) light chamber and a 30 × 30 × 30 cm^3^ dark chamber connected by a 10 × 10 cm^2^ door in the middle of the wall separating both chambers. The test was performed during five minutes. Each rat was placed in the middle of the light chamber and the latency to enter to the dark chamber, the time spent in the light chamber, the number of entries to the dark chamber, and the pokes and rearing in the light chamber were determined. The test apparatus was cleaned with 70% ethanol solution before introduction of the next animal.

#### 4.2.2. Open Field Test

The open field test was performed in an open illuminated area of 100 × 100 cm^2^ enclosed by 40 cm high walls, where the total area was divided in 25 squares of 20 × 20 cm^2^. Rats were placed in the middle of the arena and were allowed to explore over the course of 5 min. Distance travelled, rearing conduct, freezing and thigmotaxis were recorded. Thigmotaxis was calculated by measuring the time in which rats were moving adjacent to the walls of the open field chamber. The chamber was cleaned with 70% ethanol solution before the next test. Distance travelled analysis was performed using the behavioral observation research interactive software (BORIS). 

### 4.3. Tissue Processing and Immunofluorescence

Tissue processing and immunofluorescence were performed as previously reported [40,41]. Briefly, rats were anesthetized with sodium phenobarbital (120 mg/kg, i.p.) and euthanized via intracardiac perfusion with 0.9% NaCl solution, followed by 4% paraformaldehyde in PBS. Immediately, the brains were dissected, postfixed overnight in paraformaldehyde and transferred to a sucrose gradient. The ventral hippocampus (from −5.18 to −6.62 mm posterior to Bregma) was serially sectioned on a cryostat (at −21 °C), yielding slices of 20 µm. The slices were mounted on gelatinized slides and stored at −60 °C until the beginning of the immunofluorescence process. 

The brain sections were processed via immunofluorescence to evaluate the presence of the following types of cells: proliferative cells, proliferative Type 1 cells, Type 2b and 3 cells, neuroblasts and IGNs. Double labeling (KI67+/GFAP+) was employed to identify proliferative Type 1 cells, while single labeling (DCX+) was used for evaluating Type 2b and 3 cells, neuroblasts and IGNs [1]. 

For the double labeling, the coronal sections were hydrated and then incubated with antigen retrieval reagent (Dil 1:20 prewarmed at 60 °C, 858020, Bio SB) for 20 min into a mini incubator (I5110A Labnet), also prewarmed at 60 °C. Next, the slices were again incubated with the antigen retrieval reagent (Dil 1:20 at room temperature) for 20 min at room temperature. At the end of the antigen retrieval procedure, the slices were permeabilized using Triton X-100 (0.2% in PBS 1X). Subsequently, they were incubated in a blocking solution (1% bovine serum albumin, 0.1% Triton X-100, 2% horse serum) for 1 h, followed by an incubation period with a primary rabbit anti-KI67 antibody (1:50, Ab16667, Abcam) at room temperature for ~20 h. Next, a secondary antirabbit IgG-Alexa Fluor 488 antibody (1:100, A21206, Thermo Fisher Scientific, Waltham, Massachusetts, USA) was incubated for 2 h at room temperature. For the colabeling using GFAP, the brain sections were incubated with a rat anti-GFAP antibody (Dil 1:100, 130300, Thermo Fisher Scientific) and a secondary antirat IgG-Alexa Fluor 594 (Dil 1:100, A11077, Invitrogen, Waltham, Massachusetts, USA). 

For DCX labeling, the slices were treated as previously described but excluding the antigen retrieval step. The antibodies employed were a goat anti-DCX antibody (1:50, sc-8066, Santa Cruz Biotechnology Dallas, Texas, USA) at room temperature for ~20 h, and a secondary antigoat IgG-Alexa Fluor 594 antibody (1:100, A21468, Thermo Fisher Scientific), applied for 2 h at room temperature. 

All slices were mounted using Vectashield supplemented with the nuclear dye DAPI (Vector Laboratories). Fluorescent signals were detected with a fluorescence microscope (Olympus BX51) and a confocal microscope (Nikon A1R HD25). The images were processed with the Image Pro-Plus version 6.0 software (Media Cybernetics Inc., Rockville, MD, USA) or NIS Elements Version 5.20.01 Imaging Software (Nikon., Prague Czech Republic). 

### 4.4. Morphological Criteria for Cell Identification

Most of the morphological criteria have been previously described [40,41]. Proliferative cells were identified by the presence of KI67 in their cell nucleus and the nuclear location was confirmed via colocalization with marker DAPI. Proliferative-Type 1 cells were recognized by the double labeling KI67+/GFAP+ [42,43]. Based on previous findings [44], we studied two subpopulations of Type 1 cells: radial and horizontal. Radial Type 1 cells have a long dendritic process oriented vertically with respect to the granule cell layer. In contrast, horizontal type 1 cells extend their dendritic processes parallel to the granule cells layer. 

**Figure 12:**
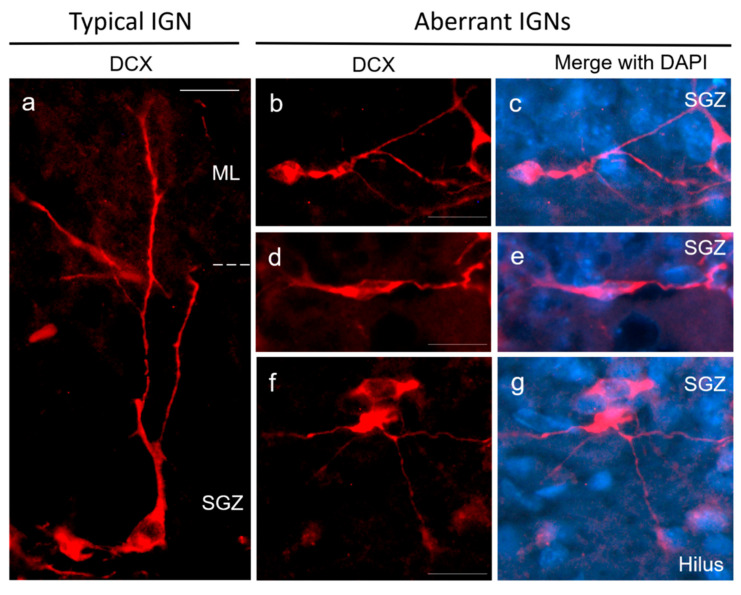
Morphologies of aberrant immature granular neurons (IGNs) located in the ventral dentate gyrus. (**a**) Typical morphology of IGNs in the ventral dentate gyrus (DCX: doublecortin). Their somas are on the subgranular zone (SGZ), and their dendrites are projected to the molecular layer (ML) of the dentate gyrus. (**b**–**g**) Aberrant IGNs with monopolar (**b**,**c**) or bipolar (**d**,**e**) dendritic processes oriented horizontally to the granule cell layer, or with dendritic processes toward the hilus (**f**,**g**). DCX: red, DAPI: blue, Scale bar: 20 µm.

The DCX marker is highly expressed during the neurogenic process by different cell subpopulations such as Type 2b and 3 cells, neuroblasts and IGNs [45,46,47]. We employed the DCX marker to aid in identifying the follow cell types: (1) Pool of Type 2b/3 cells and neuroblasts, ovoid-shaped cells lacking dendritic processes or with short plump processes [42,48]; (2) IGNs, cells that exhibit dendritic processes into the granule cell layer or long processes reaching the molecular layer of the DG; (3) aberrant IGNs, the dendritic processes of which were oriented horizontally to the granule cell layer or toward the hilus (Figure 12); and (4) ectopic IGNs, cells located in the hilus, stratum lucidum or oriens and radiatum layers. For these two last types of cells, only IGNs with second-order dendrites or more were included in the cell counting.

### 4.5. Cell Counting

**Figure 13:**
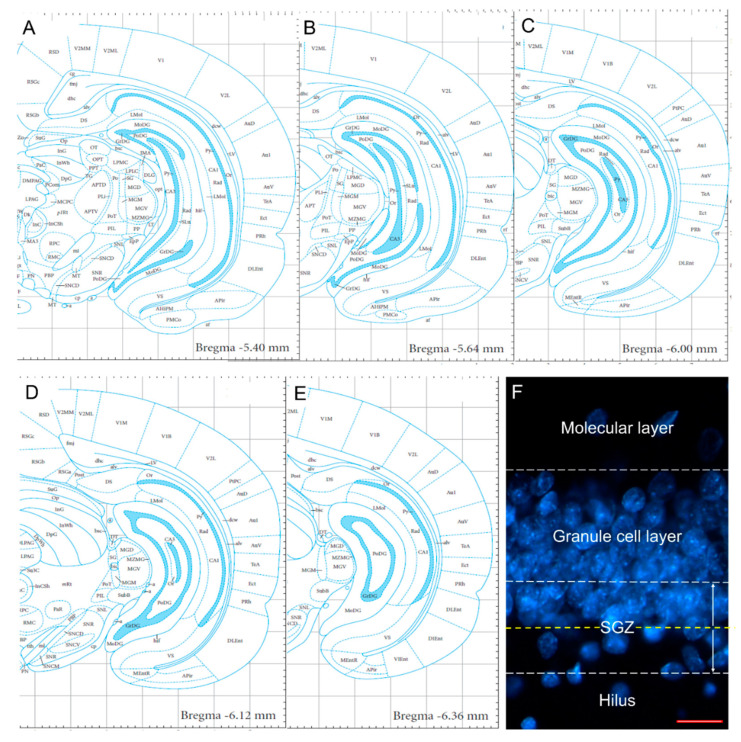
Ventral hippocampus and its subregions implicated in the study. (**A**–**E**) Schemes showing representative coronal slices of the ventral hippocampus along the rostro caudal axis; the anteroposterior coordinates are shown in the inferior part of each scheme. Moreover, the granule cell (GrDG), molecular (MoDG) and polymorphic (PoDG, also called hilus) layers of the dentate gyrus are indicated in each scheme (schemes modified from Paxinos and Watson, 2007 [49]). (**F**) Representative photomicrography evidencing the limits between molecular layer, granule cell layer, subgranular zone (SGZ) and hilus of the dentate gyrus. The granule cell layer was defined as the region constituted by the cell bodies of the granular neurons, the molecular layer refers to the area located above of the granule cell layer, and the SGZ was considered as the area occupied by two cell bodies, pointing both up and down (white dotted lines), from the inferior edge (yellow dotted line) of the granule cell layer. Scale bar (red): 20 µm.

The present study was restricted to the ventral portion of the DG of the hippocampus; this region was analyzed in slices, and cell counting was carried out in five slices per rat (the approximate coordinates were −5.42, −5.66, −5.9, −6.14 and −6.38 mm anteroposterior to Bregma (Figure 13A–E)). All cell types were counted in both brain hemispheres, and the total number of cells was calculated by adding the number of positive cells found in five slices. Data obtained from different animals within the same experimental group were averaged and shown in the graphs.

The total proliferative cells were counted in the subgranular zone (SGZ) and the hilus of the DG. Proliferative Type 1 cells, Type 2b/3 cells, neuroblasts, IGNs and aberrant IGNs were quantified in the granule cell layer/SGZ of the DG. Ectopic IGNs were counted in the hilus, stratum lucidum and oriens and radiatum layers. 

The granule cell layer was defined as the region constituted by the cell bodies of the granular neurons. The SGZ was considered to be the area occupied by two cell bodies up and down considering the inferior edge of the granule cell layer (Figure 13F). The hilus was defined as the area located between both layers of granule cells, excluding the SGZ and the portion of CA3 that enters to this region (Figure 13F) [41]. For the best reference of the other hippocampal layers (stratum lucidum and oriens and radiatum layers), consult the Paxinos and Watson Brain Atlas [49]. 

### 4.6. Western Blot

Protein extraction of whole hippocampal tissue was performed using a NP-40 lysis buffer containing protease inhibitors (Complete, Roche, Darmstadt, Germany). In brief, 30 μg of protein per condition were resolved on 10% SDS-PAGE gels, transferred to nitrocellulose membranes (BioRad, 1000 Alfred Nobel Drive Hercules, CA 94547, USA) and blocked 1 h with a 3% BSA/TBS + 0.1% tween solution. The membranes were incubated overnight at 4 °C with primary antibodies against pERK1/2, (1:4000, 9101, Cell Signaling, 3 Trask Lane Danvers, MA 01923, USA), total ERK1/2 (comprising two bands that correspond to the p42 and p44 isoforms (1:4000, ABS4, Merck, Darmstadt, Germany), GAD65/67 (1:1500, 365180, Santa Cruz Biotechnology, 10410 Finnell Street Dallas, Texas 75220, USA), BCL2 (1:2000, ab196495, Abcam, 152 Grove Street Waltham, MA 02453, USA) and β-Actin (1:5000, GTX629630, GeneTex, 6F-2, No.89, Dongmei Rd., East Dist., Hsinchu City 300, Taiwan). The membranes were rinsed with TBS/0.1% tween and incubated for 1 h with antirabbit and antimouse secondary antibodies diluted 1:10,000 (Santa Cruz Biotechnology, 10410 Finnell Street Dallas, Texas 75220, USA). Proteins were revealed via chemiluminescence (WBLUF0500, Millipore, Darmstadt, Germany) and the images were captured using a MiniBIS Pro imaging system (DNR Systems Imaging-Bio Ltd., P.O. Box 72, Neve Yamin 4492000, Israel). Densitometry analysis was performed using Image J 1.52a software.

### 4.7. Statistical Analysis

All data are expressed as mean ± SEM. The statistical analysis was carried out using GraphPad Prism^®^ version 9.0.2 and SigmaPlot 12.0. Body weight, as well as food and total fluid intake, were analyzed with a repeated-measures Two-Way ANOVA followed by a Bonferroni test. Water and sucrose preferences were analyzed using repeated-measures Two-Way ANOVA followed by a Bonferroni test comparing the water intake of the control group with the water intake observed in the Suc and Res groups, followed by comparison of the water intake of controls with the sucrose intake of the Suc and Res groups. Blood glucose, behavioral parameters, densitometric analysis and cell counting data (with some exceptions) were analyzed using One-Way ANOVA followed by a Bonferroni test for blood glucose, a Newman–Keuls test for behavioral parameters and a Tukey test for densitometric analysis and cell counting. Cell counting data of Type 2b/3 cells and neuroblasts, radial Type-1 cells, and percentages of subtypes of DCX+ cells were analyzed with a Kruskal Wallis Test, followed by a Tukey test or a Dunns’s test.

## 5. Conclusions

Our results support the absence of an anxious phenotype after sucrose intake in late adolescence, which is accompanied by an increased number of proliferative cells and aberrant IGNs in the ventral DG. The increased proliferation could be partially mediated by the ERK1/2 pathway and not by the antiapoptotic protein BCL2; however, other signaling pathways could be mediating the proliferation in long term exposure to sucrose, as in this condition, ERK1/2 signaling did not increase. These results contribute to a better understanding of the impact of sucrose in the remodeling of the nervous system through the adolescent period by providing new information related to the late adolescence and opens new questions that foster further research in this area.

## Data Availability

The data presented in this study are available in article.
